# Sub-optimality in motor planning is retained throughout 9 days practice of 2250 trials

**DOI:** 10.1038/srep37181

**Published:** 2016-11-21

**Authors:** Keiji Ota, Masahiro Shinya, Kazutoshi Kudo

**Affiliations:** 1 Laboratory of Sports Sciences, Department of Life Sciences, Graduate School of Arts and Sciences, The University of Tokyo, Tokyo, Japan; 2 Research Fellow of Japan Society for the Promotion of Science, Tokyo, Japan

## Abstract

Optimality in motor planning, as well as accuracy in motor execution, is required to maximize expected gain under risk. In this study, we tested whether humans are able to update their motor planning. Participants performed a coincident timing task with an asymmetric gain function, in which optimal response timing to gain the highest total score depends on response variability. Their behaviours were then compared using a Bayesian optimal decision model. After 9 days of practicing 2250 trials, the total score increased, and temporal variance decreased. On the other hand, the participants showed consistent risk-seeking or risk-averse behaviour, preserving suboptimal motor planning. These results suggest that a human’s computational ability to calculate an optimal motor plan is limited, and it is difficult to improve it through repeated practice with a score feedback.

Humans have an inherently variable motor system^1^,^2^,^3^. Even though humans follow the same action plan (for example, reaching out one’s arm or throwing a ball to the same target), the results of the action will vary with every trial. How humans deal with this motor output variability is a fundamental question in motor control and motor learning studies. Previous studies have shown that practice reduces motor output variability^4^,^5^,^6^,^7^,^8^,^9^. It has also been shown that humans can select the optimal movement trajectory that minimizes endpoint variance when performing goal-directed arm movements^1^,^10^,^11^. These studies suggest that the human motor system has the ability to learn and follows an optimal control policy in order to deal with the unavoidable variability.

However, it should be noted that these findings have been derived exclusively from studies using motor tasks in which the position of the target at which participants should aim by reaching or throwing movements had been overtly designated. In certain situations, such as an environment involving risk, participants need to decide on the position of the target by themselves, taking their own motor variability into account. For example, a tennis player requires spatial motor planning about where to aim the ball in a court, under the risk of hitting the ball out of bounds. In such motor planning, the target that should be aimed at is determined by the magnitude of motor variability (inherent uncertainty). In tennis, the optimal aiming point for scoring a point should be within and closer to the boundary line, as the ball bounce variance is small. Theoretically, ideal motor planning can be estimated from statistical/Bayesian decision theory, which is the mathematical model used for optimizing the expectation of gain/loss under conditions of uncertainty^12^,^13^. In this model, the expected gain/loss can be calculated by integrating a probability distribution of motor output, given a planned action, over a gain/loss function assigned to the output.

From the perspective of statistical/Bayesian decision theory, previous studies have compared ideal motor planning with actual human motor planning under conditions involving various risks. These studies have shown that human motor planning can be optimal (risk-neutral) under a symmetric configuration of the gain function^14^,^15^,^16^,^17^,^18^. However, it can be suboptimal (risk-sensitive) under an asymmetric configuration of the gain function, in which the complexity of motor planning increases^19^,^20^,^21^. The violation of risk-neutrality has also been reported in motor tasks that require choosing between 2 options that involve different variance of rewards^22^,^23^. The effect of learning on suboptimal motor planning should be elucidated, because it is not clear whether humans could learn to aim optimally by observing the results of their own motor output repeatedly, and accumulating knowledge about their own output distribution (motor variance). It is also not clear how risk-seeking (risk-seekers) or risk-averse (risk-avoiders) planners update their aiming point with a decrease in variability through repetitive practice.

To address these questions, we designed a coincident timing task that required participant motor planning about when to respond under risk, and conducted a learning experiment that lasted 9 days. By showing that suboptimal motor planning was preserved throughout the 9 days of practice, we demonstrate that it is difficult to learn the optimal aiming point by repetition and that risk-preference is retained throughout the practice period.

## Results

### Modelling of optimal motor planning

**Figure 1:**
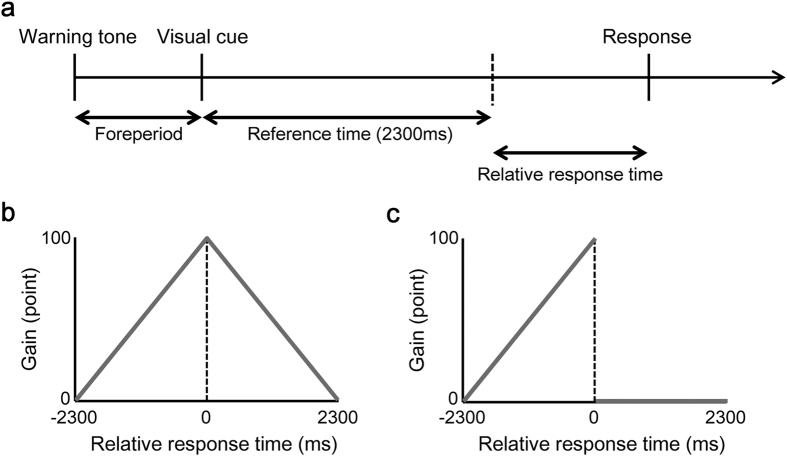
Experimental task and conditions. (**a**) The coincident timing task. After a warning tone and a random foreperiod interval, a visual cue was presented as a starting signal. The participant was required to press a button. For each trial, the participants were rewarded with a point that was determined as a gain function of the relative response time. The relative response time and the obtained point were also informed to the participants. (**b**) No Risk condition. The gain was proportional to the absolute value of the relative response time. (**c**) Risk condition. The gain was proportional to the relative response time if the participants pressed a button equal to or before the reference time (fixed to 2300 ms, this was not known to the participants), whereas no gain was obtained if the response time exceeded the reference time. In both conditions, a maximum-possible 1-trial gain of 100 points could be obtained by responding exactly at the reference time.

The participants performed a coincident timing task under risk conditions. After a warning tone was sounded as the “ready” signal and a visual cue was given as the start signal, the participants pressed a button (Fig. 1a). In each trial, they were told their relative response time (response time – reference time [fixed to 2300 ms]), the single trial gain, which was determined using the gain function, and the cumulative total gain as performance feedback. (In this study, we termed the average relative response time across trials the “mean response time”). The participants mainly performed the task under the Risk condition, which employed an asymmetric configuration of the gain function (Fig. 1c). In this condition, higher gain (100 points at maximum) was attributed when the participants pressed a button with a response time that was closer to the reference time, whereas no gain (0 points) was attributed when their response time exceeded the reference time. When the participants failed to gain a point, they were cautioned by an unpleasant alarm and a flashing red lamp on the screen.

The participants were required to make a decision about when to respond for maximizing the total gain in each block (1 block consisted of 50 trials) as best they could, and performed the task under the Risk condition for 5 blocks per day (45 blocks, or 2250 trials in total). We used a visual as well as a verbal explanation to inform the participants about the structure of the gain function, prior to performing the task.

**Figure 2:**
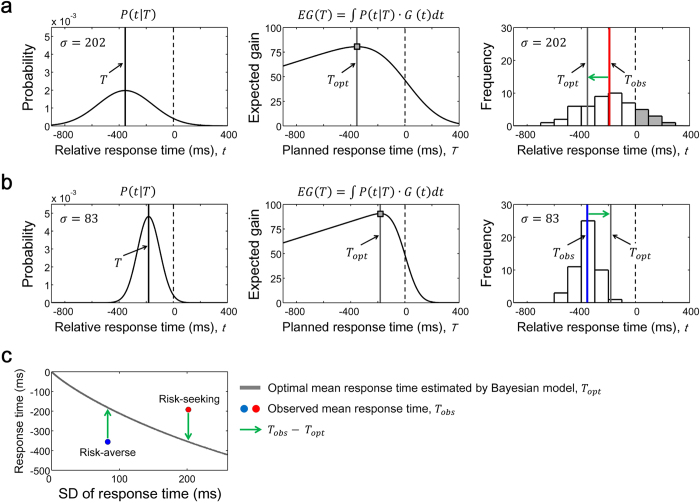
Modelling of the optimal motor plan. (**a**) The optimal strategy in the case of large response variance (σ = 202). The left panel shows the probability distribution of the executed response time. The solid black line indicates the planned response time in the probability distribution. The middle panel shows the expected gain as a function of the planned response time. The expected gain was calculated using Equation (2). The optimal mean response time *T*_*opt*_ was defined so as to maximize the expected gain (shown as a solid grey line). The right panel shows the actual histogram of the executed response time from a single participant. The observed mean response time *T*_*obs*_ (shown as a solid red line) is closer to the reference time than the optimal response time *T*_*opt*_; therefore, this strategy is classified as risk-seeking, as it leads to too many error trials, shown in the filled bars. A 1-way green arrow shows the difference between *T*_*obs*_ and *T*_*opt*_. (**b**) The optimal strategy in the case of small response variance (σ = 83). The middle panel shows that the optimal mean response time in the case of small variance is closer to the reference time than that in the case of large variance. The right panel also shows the actual histogram from a single participant. In this case, the observed mean response time (shown as a solid blue line) is further from the reference time than the optimal time; therefore, this strategy is classified as a risk-averse strategy. (**c**) The panel shows that the optimal strategy depends on response variance. The optimal mean response time (shown as a grey curve) is located closer to the reference time, as the SD of the response time is smaller. If the observed mean response time was located in line with this curve, it could be proposed that participants behaved optimally given their own response variance. This example shows that the risk-seeking and risk-avoiding participants took higher (shown as a red circle) or lower (shown as a blue circle) than optimal risks, respectively.

Based on the statistical/Bayesian decision theoretical approach^14^,^15^,^16^,^17^,^18^,^19^,^20^,^21^, we estimated the optimal mean response time in each experimental block by integrating the gain function over the probability distribution of the executed response time and compared it with participant’s actual mean response time. The probability distribution of the executed response time followed a Gaussian distribution for a given planned response time and response variance (Fig. 2a,b left panel). Given the particular magnitude of the response variance, we could calculate the expected gain as a function of the planned response time and defined the optimal mean response time *T_opt_* for maximizing the expected gain (Fig. 2a,b, middle panel). Under the Risk condition, the optimal mean response time is located closer to the reference time, as the response variance is smaller (Fig. 2c). If the observed mean response time *T^obs^* corresponded to the optimal mean response time, it indicated a risk-neutral strategy for a given response variance. On the other hand, if it were located closer to or further from the reference time than the optimal value, the participant was adopting suboptimal risk-seeking (Fig. 2a, right panel) or risk-averse strategies (Fig. 2b, right panel). As an index of the optimality of motor planning, we calculated the difference between the observed mean response time *T*_*obs*_ and the optimal mean response time *T*_*opt*_, as shown in Fig. 2a,b, right panel, and Fig. 2c. Therefore, the way to improve the expected gain was to reduce the response variance and to learn the optimal motor plan for one’s own magnitude of response variance.

### Effect of learning on response variance and motor planning

**Figure 3:**
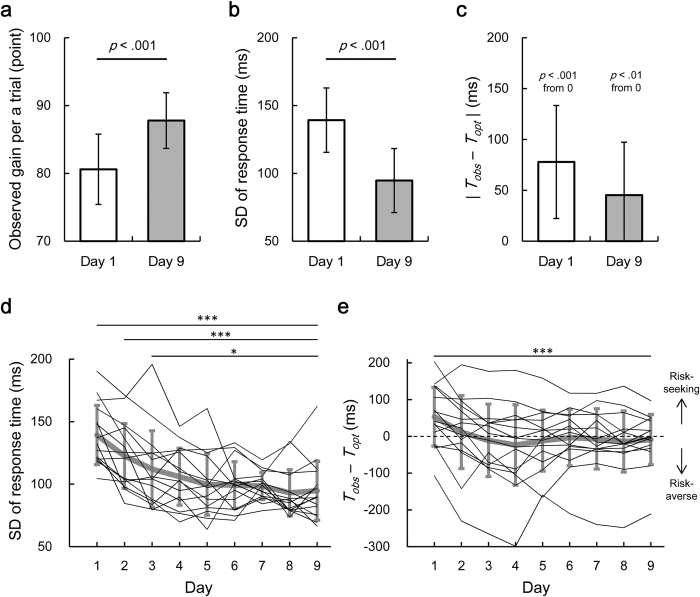
Effect of learning on performance indexes. (**a**) Observed gain per trial. (**b**) Standard deviation (SD) of the response time. (**c**) Absolute difference between the observed mean response time *T*_*obs*_ and the optimal mean response time *T*_*opt*_. The average value across the participants is plotted in each index, and the error bars indicate the standard deviation. The observed gain per trial and the SD of the response time was significantly improved from day 1 to day 9. The absolute difference between *T*_*obs*_ and *T*_*opt*_ was significantly different from 0 (risk-neutrality value) both on day 1 and day 9. Thus, the participants still behaved sub-optimally on the last day. (**d**,**e**) Time series of the SD of response time (**d**) and the difference between *T*_*obs*_ and *T*_*opt*_ (**e**), shown for all the participants. The thin black lines represent the average value on a day (5 blocks) for each participant. Intra-personal errors are omitted for clarity. The thick grey line represents the average value across the participants and the error bars indicate the standard deviation. *indicates *p* < 0.05 and ***indicates *p* < 0.001, compared to day 9 (Dunnett’s post-hoc test following one-way repeated measures ANOVA).

We first analysed the improvement in performance indexes induced by practice. The expected gain earned by the participants significantly increased from day 1 to day 9 (*t* [14] = −5.78, *p* < 0.001, *d* = −1.49, Fig. 3a). The SD of the response time as an index of response variance also decreased from day 1 to day 9 (*t* [14] = 9.12, *p* < 0.001, *d* = 1.82, Fig. 3b). Therefore, we confirmed that the participants obtained a smaller response variance with practice, and this resulted in the increase of the observed gain per trial. On the first day of the practice, we investigated whether the absolute difference between the observed and the optimal mean response time differed from the risk-neutrality value (i.e., 0), by a 1-sample *t*-test, and found it to be significantly different (*t* [14] = 5.25, *p* < 0.001, Fig. 3c). We also found that it was significantly different from 0 on the last day of practice (*t* [14] = 3.26, *p* < 0.01, Fig. 3c). These results suggested that the participants showed suboptimal risk-sensitive behaviour on day 1 and still did on day 9.

We next focused on a time series of the response variance and the difference between *T*_*obs*_ and *T*_*opt*_. The averaged values on a day (5 blocks) for both indexes are plotted in Fig. 3d,e for all the participants (thin black lines). The averaged value across the participants (thick grey line) is also plotted. Dunnett’s post-hoc test after one-way repeated measures ANOVA showed that there were significant differences in the SD of response time on days 1, 2, and 3, compared to that on day 9 (one-way ANOVA: *F* [8, 112] = 15.40, *p* < 0.001, Dunnett’s test: *ps* < 0.05, Fig. 3d). Thus, the decrease in the SD of the response time peaked at day 4, and plateaued thereafter. This is a general tendency in motor learning: rapid learning occurs at the beginning of a practice, and it becomes more gradual later on^24^. If the practice could improve the risk-sensitive behaviour observed on day 1, the difference between the observed mean response time *T*_*obs*_ and the optimal mean response time *T*_*opt*_ would converge toward 0 (risk-neutrality). However, no such trends were found in each participant (thin black lines, Fig. 3e). In general motor learning tasks in which the position of the target at which participants should aim is designated by experimenters, the large constant error (i.e., a difference between the target and a motor response) emerges in the initial trial. By observing this error, the motor response gradually converges toward the target, and the constant error undershoots or overshoots crossing 0 thereafter^25^. However, in our task (requiring a motor plan for aiming point), the value of the difference between *T*_*obs*_ and *T*_*opt*_ remained positive (risk-seeking) or negative (risk-averse). This would be one feature of learning during the motor planning under risk. Overall, compared to the difference between *T*_*obs*_ and *T*_*opt*_ on day 9, we only found it to be significantly smaller than that on day 1 (one-way ANOVA: *F* [8, 112] = 7.11, *p* < 0.001, Dunnett’s test: *p* < 0.001 Fig. 3e).

### Consistency of motor planning under risk

**Figure 4:**
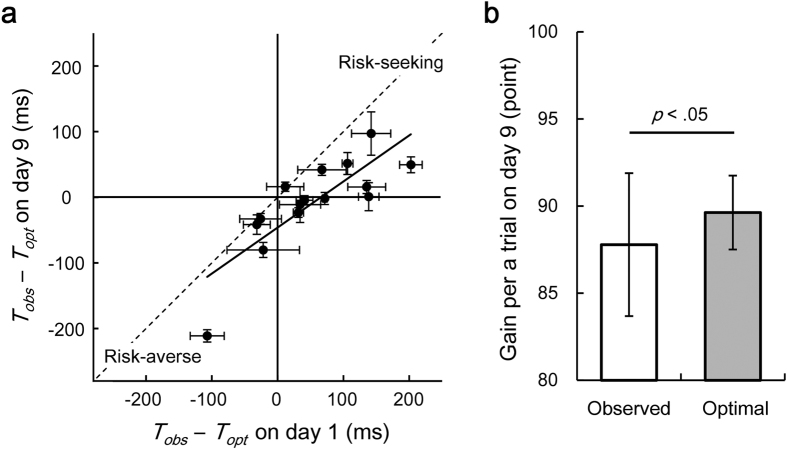
Suboptimal behaviour was preserved after the practice. (**a**) The average difference between the observed mean response time *T*_*obs*_ and the optimal mean response time *T*_*opt*_ on day 1 (5 blocks) is plotted against that on day 9 for all the participants. Horizontal and vertical error bars indicate the standard errors of the mean within day 1 and day 9, respectively. Data points that fall in the top right quadrant correspond to the risk-seeking to risk-seeking strategy. Data points that fall in the bottom left quadrant correspond to the risk-averse to the risk-averse strategy. A slope of 0.70 in a regression line suggests that the participants kept risk-seeking or risk-averse strategy between day 1 and day 9. (**b**) The suboptimal strategy was still used on the last day. On day 9, there was a significant difference between the observed gain and the optimal gain per trial. The average value across the participants and the standard deviation is plotted.

Figure 4a shows the average difference between the observed and the optimal mean response time on day 1 against that on day 9 for all 15 participants. If the participants switched the strategy from risk-seeking to risk-averse and from risk-averse to risk-seeking, the slope of the regression line between day 1 and day 9 would be negative. If all the participants would take a risk-neutral strategy by day 9, the slope would be 0. A least-squares linear regression resulted in a slope of 0.70 (95% confidence interval of the slope: 0.41–0.998, *R*^2^ = 0.67, *p* < 0.001), suggesting that the individual participants retained their risk-seeking or risk-averse strategies. This consistently held true when we performed the same linear regression analysis between other dates from day 1 to day 9 (Supplementary Table 1). The linear regression between other dates resulted in a mean slope of 0.81 ± 0.14.

On the last day of the practice, the observed gain per trial was significantly smaller than the optimal gain that the participants could theoretically obtain (*t* [14] = −2.69, *p* < 0.05, *d* = −0.54, Fig. 4b). Thus, as shown in Fig. 3c, the participants still showed suboptimal risk-sensitive behaviour on day 9, and this yielded a reduced observed gain, as compared to the optimal gain. These findings suggest that the effect of learning on motor variability is effective, but it is difficult to learn the optimal motor plan for the given individual’s motor variability, even over a practice period of 9 days.

Previous studies have confirmed risk-sensitive behaviour in a motor task and have explained the reason from the basis of the prospect theory^19^,^22^,^26^ which proposed that individual risk-sensitivity can be explained by a distortion of utility function^27^. Here we explained the participant’s behaviour by introducing the utility function, similar to the study by O’Brien & Ahmed^19^. The utility of the reward (subjective reward) is represented by the power function of the actual reward^28^, *U*(*x*) = *x*^*α*^. The participant’s specific exponential parameter *α* represents their risk-sensitivity. In our study, the participants with a convex utility function (*α* > 1) would likely respond closer to the reference time (risk-seeking behaviour). In contrast, the participants with a concave utility function (*α* < 1) would likely respond further from the reference time (risk-averse behaviour). We estimated the value of *α* for each day and each participant by fitting the observed mean response time data in a day (5 blocks) to the subjective optimal mean response time that maximizes the expected utility.

**Figure 5:**
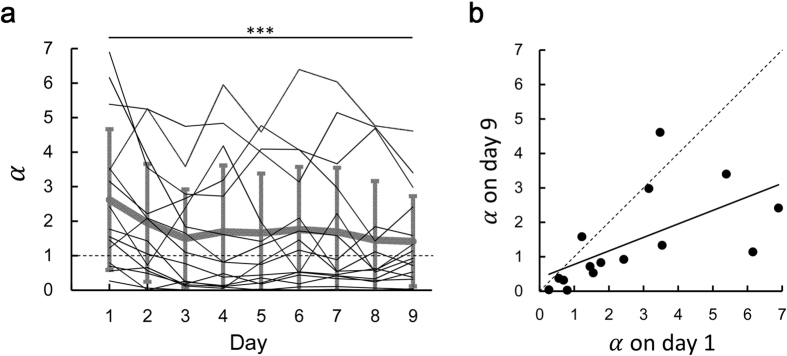
Estimation of the utility function. (**a**) Time series of the estimated values of *α*. The thin black lines represent the value of *α* on a day for each participant. The thick grey line represents the average value across the participants and the error bars indicated the standard deviation. ***indicates *p* < 0.001 compared to day 9 (Dunnett’s post-hoc test following one-way repeated measures ANOVA). (**b**) The value of *α* on day 1 is plotted against that on day 9. Each circle represents each participant.

In Fig. 5a, we plotted the estimated value of *α* in each participant (thin black lines) against practice dates. A thick grey line represents the averaged value across the participants. Eleven of 15, nine of 15, and seven of 15 participants had a convex utility function on day 1, day 2, and day 9, respectively (mean *α* = 2.62 ± 2.04, 1.95 ± 1.71, and 1.41 ± 1.31). Dunnett’s post-hoc test following one-way repeated measures ANOVA showed that there was the significant difference in *α* only on day 1, as compared to *α* on day 9 (one-way ANOVA: *F* [8, 112] = 2.99, *p* < 0.05, Dunnett’s test: *p* < 0.001 Fig. 5a), suggesting that the change in the utility function reached a plateau at day 2. We next performed linear regression analysis. A least-squared linear regression between *α* on day 1 and *α* on day 9 resulted in a slope of 0.39 (95% confidence interval of the slope: 0.09–0.70, *R*^2^ = 0.37, *p* < 0.05, Fig. 5b). We performed the same regression analysis between other dates from day 1 to day 9 (Supplementary Table 2). A linear regression resulted in a mean slope of 0.79 ± 0.19. The findings indicated that the participants showed a consistent trend in the direction of the distortion in the utility function (convex or concave function).

**Figure 6:**
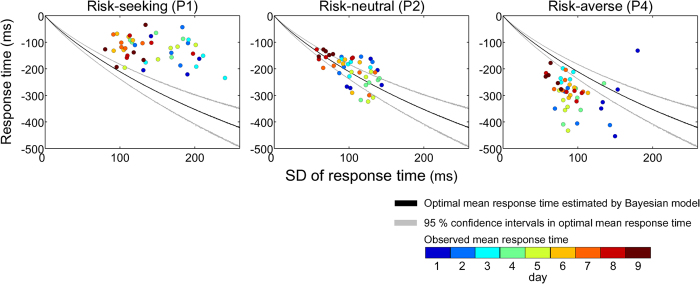
Inter-personal differences in strategy under risk. Each panel shows a participant classified as risk-averse, risk-neutral, and risk-seeking. In each panel, the observed mean response times for all 45 blocks were plotted against the SD of the response time. The colour scale of the circles indicates the day of the measurements. Black curves indicate the optimal mean response time calculated by the Bayesian model (Equation 2). Grey curves show the 95% confidence intervals of the optimal mean response times after 3000 bootstrap replications. See Methods and Ota *et al*.^20^ for the details of the calculations. The risk-seeking participant (left panel) responded closer to the reference time than the optimal time, and the risk-averse participant (right panel) responded further from the reference time than the optimal time. The risk-neutral participant (middle panel) could appropriately increase their mean response time with a practice-induced decrease in the response variance. For the results of the other participants, see Supplementary Fig. 2.

We next showed the various transition patterns of the observed mean response time for participants with a different strategy. In Fig. 6, we plotted the observed mean response times for all 45 blocks (circles), the optimal mean response time (black curve), and its 95% confidence intervals (grey curves) against the SD of the response time. As shown in Fig. 6, the SD of the response time gradually decreased as the practice proceeded, while the observed mean response times were distributed differently. In participant P1 (risk-seeking), the observed mean response times were closer to the reference time than the upper limit of the 95% confidence interval (Fig. 6, left panel). This participant consistently had a response time close to the reference time, regardless of the magnitude of the response variance. In contrast to P1, participant P4 (risk-averse) had response times that gradually became closer to the reference time, with a decrement in the response variance, but with the observed mean response times further from the reference time than the lower limit of the 95% confidence interval (Fig. 6, right panel). These participants took lower or higher than optimal risks, given their own variance in motor output. For participant P2 (risk-neutral), the observed mean response times were mostly distributed within the Bayesian-theoretical 95% confidence interval (Fig. 6, middle panel). This participant had response times that gradually approached the reference time, with a decrease in the response variance for taking optimal risks. Thus, there are different transition patterns in aiming for a given current motor variability. For the results of the other participants, see Supplementary Fig. 2.

To investigate the optimality of motor planning under a symmetric gain function, we also tested the No Risk condition, in which the gain was also achieved if the participants responded after the reference time, and it was proportional to the absolute value of the relative response time (Fig. 1b). The participants performed this condition for 100 trials on day 1 and day 9. Under the No Risk condition, we confirmed that there were no significant differences (*t* [14] = 1.26, *p* = 0.23, *d* = 0.46) between the optimal (Mean = 0 ± 0 ms) and the observed mean response time on day 1 (Mean = 18.86 ± 57.92 ms). This result verified that the suboptimal motor planning found under the Risk condition was due to the asymmetric configuration of the gain function, in accordance with the results found in our previous study^20^.

In Equations 1 and 2, we modelled the optimal mean response time based on the assumption that temporal variance is constant regardless of the magnitude of planned response time. According to Weber’s law, the standard deviation (SD) of response time could be proportional to the magnitude of the planned response time^29^. Here we calculated the optimal mean response time based on the model that takes Weber’s law into account. We found that there was a marginal difference between these two models, suggesting that the issue of Weber’s law (signal-dependent noise) would not jeopardize our argument (for detail, see Supplementary Fig. 3).

## Discussion

Sub-optimality in motor planning and motor control has been investigated over the last decade^19^,^20^,^21^,^22^,^23^,^26^,^30^. For example, Wu *et al*.^21^ investigated the mean endpoint of pointing movements under both a symmetric and an asymmetric expected-gain landscape. They found that participants performed optimally under a symmetric expected-gain landscape in which the optimal endpoint lay within the target region, whereas the same participants performed sub-optimality under an asymmetric expected-gain landscape in which it lay within the penalty region. O’Brien & Ahmed^19^ used a gain function in which a higher gain was accompanied by risk of zero or negative gain. Under such an asymmetric configuration, suboptimal risk-seeking behaviour was confirmed in reaching tasks and whole-body movement tasks^19^. Our previous study also exploited a similar gain function for a coincident timing task and confirmed that humans tend to take higher risk than optimal under the asymmetric gain function, regardless of the location of the penalty region^20^.

A question that arose from these previous studies was related to the trainability of the motor decision. In other words, it has been unclear whether humans could update their motor planning to achieve maximum expected gain by observing the total gain of an experimental block and by repeating blocks many times. In this study, we found that ten of fifteen participants behaved sub-optimally even after 9 days or 45 blocks of practice. The SD of response time changed until day 4, while the difference between the observed mean response time and the optimal mean response time, or the distortion of the utility function changed until day 2. The participants showed consistent risk-sensitive behaviour (adopting a risk-seeking or risk-averse strategy) throughout the practice. Thus, humans contradict a Bayesian decision maker who can calculate the optimal strategy for any combination of gain/loss function, as well as the probability distribution of the motor output^14^. Taken together, our findings indicate that it is relatively difficult to improve the computational ability for planning an optimal aiming point through repeated practice.

Motor learning studies have exploited motor tasks in which the position of the target at which participants should aim was clearly designated. In these situations, humans can learn a less variable motor response to a target^4^,^5^,^6^,^7^,^8^,^9^ and can also adapt movement to reach a target under a novel environment, with perturbation produced by visuo-motor rotation or force field^31^,^32^,^33^,^34^,^35^,^36^,^37^. The basic model of motor learning proposes that learning occurs by updating the internal model based on an error (discrepancy) between the target and the resulting motor output in a trial-by-trial basis^32^,^35^,^37^. Humans can adapt to a novel environment and reach a particular target within 100 trials^33^,^35^.

In our task, a target (an aiming point) was not designated and had to be learned. Thus, an error against the explicit target was not shown to the participants in trial-by-trial manner. Instead, they were required to update their motor planning (an aiming point) based on a distribution of motor output in a block-by-block manner. Our results suggested that 45 blocks (2250 trials) were not sufficient to establish such learning of an aiming point. Knowledge of the motor output distribution is accumulated by observing the result of the output in a trial-by-trial basis. In our task, therefore, the participants needed to update their aiming point (mean response time), based on the observed distribution among blocks, while compensating for the error between their implicit aiming point and their result of motor output in a trial-by-trial manner. In addition, because the optimal aiming point depends on motor variability, it can change with the reduction of variance caused by repetition of trials. For these reasons, the learning of an aiming point can be difficult and requires many trials.

Why did participants persist in adopting a suboptimal risk-seeking/risk-averse strategy? In the statistical/Bayesian decision-making model, integration of the product of gain function and motor variability determines the expected gain and optimal motor plan (Equation 2). If the subjective evaluation of these two functions was distorted from objective reality, the equation would yield a suboptimal motor plan. In the field of behaviour economics, prospect theory^27^ and cumulative prospect theory^38^ have proposed that suboptimal economic decisions are due to a distortion of the utility from the actual gain/loss and a distortion of the probability weighting from the actual probability. Previous studies have confirmed risk-sensitive behaviour in a motor task and have explained the reason from the basis of the prospect theory^19^,^22^,^26^. Based on these studies, we will discuss two possible reasons for risk-seeking/risk-averse behaviours in below.

One would be a distortion in the utility of the gain function^19^. In the study of O’Brien & Ahmed^19^, participants over-weighted point rewards and under-weighted point penalties under a similar gain function as in the Risk condition, in which higher gain comes with the penalty (called the “cliff paradigm”). They showed that most participants adopted risk-seeking behaviour and suggested that this subjective weighting of rewards/penalties could be one reason for risky planning. In contrast, risk-averse decisions could arise from a concave utility function, which indicates a decaying utility in comparison to the actual reward^28^. In this study, we also estimated the participant’s utility function of the rewards from their actual planning data, and found that the participants showed a consistent trend in the direction of the distortion (concave or convex utility function). A further reason may be that risk-avoiders may be discouraged from responding late under Risk condition due to a penalty. In this study, the participants received a penalty involving an unpleasant alarm if they achieved zero gain. Although the volume of the alarm was the same for all participants (see Methods), the utility of the penalty may be different between them, which also possibly influenced individual motor planning.

A second possibility would be a distortion in the probability weighting of one’s own motor output^19^,^22^,^26^. Despite the importance of possible motor errors of one’s own upcoming action (prospective error) in motor learning^39^, the subjective assessment of the prospective error may be unreliable. In the study of Nagengast *et al*.^22^ and Wu *et al*.^26^, participants under-weighted small probabilities and over-weighted moderate to large probabilities when required to point to either 1 of 2 targets, in which the hit probabilities differed. Most participants exhibited risky choice behaviour^22^,^26^ because these distortions led participants to believe that they had smaller movement variability than they actually had^19^. These researchers have also shown that some participants show an opposite distortion pattern (overweighting small probabilities and underweighting moderate to large probabilities). Therefore, these studies suggest that risk-seekers in our study would underestimate their own response variance, whereas risk-avoiders would overestimate their own variance. The underestimation of motor variance was also seen in a temporal task^40^ as well as in a spatial task. In our experiment, however, we could not determine which distortions (rewards/penalties or probabilities) mostly affected participant’s behaviour, because we could only estimate one parameter. This point should be investigated further in future.

Importantly, suboptimal behaviours were still observed on the 9th day. This indicated that an inappropriate representation of one’s own variance might be retained throughout the practice. A previous study has also confirmed the evidence of an inaccurate internal model of own motor error after extensive training^41^. In the study by Zhang *et al*.^41^, participants assumed their own endpoint variance in the reaching task to be isotropic distribution, rather than the actual anisotropic distribution, even after hundreds of trials. In other situations, participants represented their visuo-motor error distributions as a mixture of small (2–6) sets of non-overlapping uniform distributions, rather than a single Gaussian distribution, even though their actual motor errors closely approximated a Gaussian distribution^42^. In our experiment, the participants performed the timing task for 2250 trials in total. Taken together, human probability interpretation can be distorted from the true distribution in sensorimotor output, and this inaccurate interpretation is difficult to improve solely by observing the motor outputs repeatedly.

Recent studies have revealed brain areas related to components of statistical/Bayesian decision theory (i.e., gain function and motor uncertainty). For example, it has been suggested that broad regions, including the ventromedial prefrontal cortex (vMPFC)/the oribitofrontal cortex (OFC), the ventral striatum, and the insula, are involved in value representation in the human brain (for review, see Bartra *et al*.^43^; Levy & Glimcher^44^; Peters & Büchel^45^). Particularly, Levy & Glimcher^44^ and Peters & Büchel^45^ showed that the vmPFC/OFC is the overlapping region that represents subjective value across different types of rewards, by conducting a meta-analysis. These findings suggest that vmPFC/OFC reflects the distortion of the utility from the actual rewards.

The dorsal premotor cortex (PMd) and the primary motor cortex (M1) have been reported to contribute to motor uncertainty. It has been demonstrated that, in nonhuman primates, movement variability arises from variation in neural activity in PMd and M1^46^,^47^. How does the brain represent such motor uncertainty? Wu *et al*.^48^ showed that the probability weight is represented in the medial prefrontal cortex (mPFC) when probability information is implicit in the participant’s own motor noise and needs to be estimated. In our task, an accurate estimation of motor uncertainty was needed to calculate the Bayesian optimal solution. For instance, confounding the 4% error trials (i.e., responding after the reference time) with 3% resulted in a difference of 57 ms in the risk-neutral response time (calculated by simulation, data not shown). The brain may need much time to estimate the prospective error with such accuracy.

By integrating the gain function and motor uncertainty, the expected gain and optimal motor plan were determined in the statistical/Bayesian decision-making model. Little is known about the neural representation involved in the integration of these 2 sets of information. Ting *et al*.^49^ investigated the neural mechanisms underlying Bayesian integration using a decision-making task in which participants were required to estimate the reward probability by combining past experience (prior knowledge) with current sensory information (likelihood), and showed that the mPFC plays a role in this Bayesian integration. In future studies, it would be interesting to investigate how the brain integrates a gain function over inherent motor uncertainty and how the brain makes a suboptimal motor decision.

The evidence that humans can select a movement trajectory that minimizes task-relevant movement variance^1^,^10^,^11^ suggests that the motor system properly estimates its own motor variance. However, we observed inter-personal differences in the transition patterns of the observed mean response time. As shown in Fig. 6, participant P1 (risk-seeking) demonstrated a response time that approached the reference time rather than the optimal time, from the beginning to the end of the practice. We also found such a tendency in participants P3, P8, and P13 (Supplementary Fig. 2). These participants may use a motor heuristic approach^21^, in which the best strategy is always to aim closer to the maximum gain rather than to change the motor planning according to their own response variance. On the other hand, P4 (risk-averse) moved the mean response times closer to the reference time with a decrease in the response variance. This participant might consider his/her own variance, but the observed mean response times were located below the Bayesian-theoretical 95% confidence interval. Again, we found such a tendency in participants P7, P12, and P14 (Supplementary Fig. 2). Participant P2 (risk-neutral) almost appropriately changed the mean response time with the decrease in the response variance. We also confirmed that these inter-personal differences were not due to levels of motivation, concentration, and sleepiness (see Supplementary Fig. 1). Previous studies that had investigated motor planning under risk have shown no evidence that participants changed the mean endpoint of reaching or pointing movements, but these studies involved short periods of time (3 days at most)^16^,^17^,^19^,^21^. Thus, the current study presents novel evidence of individual differences in the transition of aiming in the presence of variability in motor output.

These individual differences indicate the importance of coaching in the sports field. For example, it would be important to teach risk-avoiders to adopt more risk-seeking behaviour (in tennis, the proper instruction would be to aim to hit an ace more aggressively, rather than to aim safely within the court). In the long run, for risk-seekers, it would be important to wait until their response variance has decreased, rather than to teach them to avoid risk (in tennis, this would mean to wait for development of the skill level, even though the individual frequently loses a point by aiming for an ace when the skill level is still poor). Actually, risk-seeking behaviours are seen in extensively trained players^50^,^51^. For example, professional NBA basketball players tend to attempt a consecutive 3-point shot after making a successful 3-point shot more often than after failing to do so, even though the chance of making a further 3-point shot successfully and the average number of points gained by an offensive team is decreased^50^. Therefore, for coaches and trainers, it is important to evaluate each player’s strategy under risk as well as their skill level.

## Methods

### Participants

This study was approved by the Ethics Committee of the Graduate School of Arts and Sciences, the University of Tokyo, and the methods were carried out in accordance with the approved guidelines. All the participants signed informed consent. Fifteen healthy right-handed adults (12 male, 3 female; mean age: 21.3 ± 3.9 years) participated in the experiment. All the participants were unaware of the purpose of the experiment.

### Experimental task and procedure

In this study, to control for an energetic cost that could possibly disturb learning of optimal planning, we used a coincident timing task that requires participants to use a constant energetic cost, whatever the planned strategy. We performed this experiment based on our previous study^20^. We show the time sequence of our basic experimental task in Fig. 1a. First, a warning tone was presented to prepare the participants to get ready for an upcoming trial. After a random foreperiod interval, a visual cue was presented on a computer screen as the start signal (14 inches, 1600 × 900 pixels, refresh frequency 60 Hz). The reference time was set at 2300 ms after the presentation of the visual cue.

In the Risk condition, when participants responded after the reference time and scored zero points, they were immediately cautioned by an unpleasant alarm that was sounded through their headphones (MDR-Z150, SONY, Tokyo, Japan) and a flashing red lamp on the screen. The volume of the alarm was 68.1 ± 0.8 dB. We defined these eventualities as an error trial and any other trials as successful trials. Of note, all the trials resulted in success in the No Risk condition, because the range of scoring more than zero points was sufficient (i.e., it was 4600 ms). We did not use actual monetary rewards/penalties; as we previously described, small amounts of monetary rewards/penalties appear to have less influence on motor planning^20^. For detailed information on the experimental task and procedure, see Ota *et al*.^20^.

### Model assumptions

We calculated the optimal mean response time that maximizes the expected (total) gain using a statistical/Bayesian decision theoretic approach^14^,^15^,^16^,^17^,^18^,^19^,^20^,^21^. Our model consisted of 2 sets and 2 functions. The 2 sets were: possible response strategy *T* (motor planning) and the executed relative response time *t* (motor output). The 2 functions were: probability distribution of the executed response 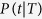 and the gain function *G*(*t*). For a planned a particular response time *T*, a particular relative response time *t* is stochastically executed due to uncertainty in the motor output. In our previous study, we confirmed that this probability distribution in a coincident timing task followed a Gaussian distribution^20^. Thus, 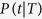 can be written as follows; this function is described in Fig. 2a,b (left panels).


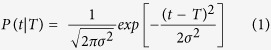


Then, for execution in a particular response time, the gain is given according to the gain function *G*(*t*). The expected gain *EG*(*T*), as a function of the planned response time *T*, can be calculated by integrating 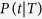 over *G*(*t*) as follows; this function is also described in Fig. 2a,b (middle panels).


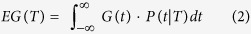


We can calculate the optimal mean response time *T*_*opt*_ by maximizing Equation (2). Under the Risk condition, the optimal mean response time *T*_*opt*_ depends on the magnitude of the response variance *σ* (visualized in Fig. 2c as a solid curve). A Bayesian decision maker chooses a response time *T*_*opt*_ for any given gain function *G*(*t*) and response variance *σ*^14^. We can regard *T*_*opt*_ as theoretically optimal risk-neutral planning.

We then compared the participant’s actual planning with the Bayesian optimal planning. The participant’s actual planning *T*_*obs*_ can be assumed as their mean response time in a response histogram (described in Fig. 2a,b, right panels). As an index of optimality of motor planning, we calculated the difference between the observed mean response time and the optimal mean response time for each participant and each block. Furthermore, we estimated the 95% confidence interval of the optimal mean response time using a bootstrap algorithm (visualized as grey curves in Fig. 4). We simulated 50 trials of execution of a task within a certain optimal mean response time *T*_*opt*_, with its response variance, 3000 times and obtained bootstrap samples. After we calculated the average of 1 sample in each bootstrap sample and sorted the 3000 average samples in ascending order, we defined a 2.5% and 97.5% point in the average samples as the 95% confidence interval within that optimal mean response time. For more detail about the procedure, see Ota *et al*.^20^.

We also explained the participant’s suboptimal risk-sensitive behaviour by introducing the utility function. Similar to the model used by O’Brien & Ahmed^19^, we calculated the expected utility *EU*(*T*) as a function of the planned response time *T*, as follows.


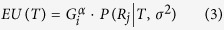


In this model, 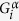 represents the subjective gain. 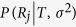 represents the probability that the executed response time falls in the reward region 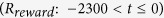 or the penalty region 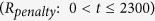, for the given planned response time and response variance. The exponential parameter *α* represents participant’s risk-sensitivity. A specific point reward *G*_*i*_ was defined by the following equation.


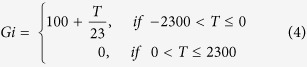


The subjective optimal mean response time was calculated by maximizing Equation (3). We estimated the value of *α* for each day by fitting the observed mean response time data in 5 blocks to the model prediction. We used the *fminsearch* function in MATLAB to find *α* that minimized the mean squared error between them. To verify that we had searched for the global minimum, we repeated the search process using different starting points.

### Practice protocol

We conducted 5 experimental sessions per day. In each experimental session, the participants firstly performed the task under the Risk condition for 1 block (50 trials). After the task, they then scored their current motivation, concentration, and sleepiness using a visual analogue scale (VAS). Lastly, they took 2–3 min rest to minimize fatigue. They moved to the next experimental session after this rest. The participants repeated this experimental session for 5 times per day. In addition to the Risk condition, they performed the task under the No Risk condition for 2 blocks (100 trials each) on the first day and on the last day.

The participants practiced the task approximately 3 times a week for 3 consecutive weeks. The average inter-practice interval was 2.28 ± 0.21 days across the participants (1 day at minimum, 6 days at maximum). This variability of the inter-practice interval did not influence the individual learning rate. There was no significant correlation between the inter-practice interval and the difference between the expected gain in the Risk condition on day 1 and that on day 9 (*r* = −0.21, *p* = 0.46, *df* = 15).

### Data analysis

For each trial, we recorded the response time (button press time – onset of a visual cue) and calculated the relative response time (response time – the reference time). We could not obtain the trials conducted for the first block on day 1 in the Risk condition for P1 and P8. Thus, we analysed the remaining 44 blocks for these. Trials in which the response times exceeded ±2.5 SD from the mean were excluded from the analysis as outliers. This resulted in the removal of 510 of a total of 33650 trials (1.5%) in the Risk condition and 55 of a total of 3000 trials (1.8%) in the No Risk condition. *P* < 0.05 was regarded as indicating statistical significance. Cohen’s *d* measure was calculated as an index of effect size.

## Additional Information

**How to cite this article**: Ota, K. *et al*. Sub-optimality in motor planning is retained throughout 9 days practice of 2250 trials. *Sci. Rep.* **6**, 37181; doi: 10.1038/srep37181 (2016).

**Publisher’s note**: Springer Nature remains neutral with regard to jurisdictional claims in published maps and institutional affiliations.

## Supplementary Material

Supplementary material
