# Supplementary material for: Sub-optimality in motor planning is retained throughout 9 days practice of 2250 trials
Source: Sci Rep. 2016 Nov 21;6:37181. doi: 10.1038/srep37181 (PMC5116677; doi:10.1038/srep37181)
Supplement: Supplementary material [file srep37181-s1.pdf]

Supplementary Material

Sub-optimality in motor planning is retained throughout 9 days practice of 2250 trials

Keiji Ota[1, 2]*, Masahiro Shinya[1], Kazutoshi Kudo[1]*

[1] Laboratory of Sports Sciences, Department of Life Sciences, Graduate School of Arts and Sciences, The University of Tokyo, Tokyo, Japan.

[2] Research Fellow of Japan Society for the Promotion of Science, Tokyo, Japan.

* Corresponding authors:

Keiji Ota

Email: keiji.o.22@gmail.com

Kazutoshi Kudo

Email: kudo@idaten.c.u-tokyo.ac.jp

1. Visual analog scale

To establish whether participant's motivation, concentration, and sleepiness influenced risk-sensitivity, we conducted a correlation analysis. The levels of 3 scales were evaluated by VAS. The correlation analysis showed that there was no significant relationship between the average difference of the observed mean response time T_{obs} and the optimal mean response time T_{opt} in all 45 blocks and the average motivation (r = - 0.10, p = 0.73, df = 15), concentration (r = 0.09, p = 0.75, df = 15), and sleepiness (r = 0.13, p = 0.64, df = 15). Therefore, inter-personal differences in strategy under risk (shown in Fig. 5 & Supplementary Fig. 2) were not due to the differences in motivation, concentration, and sleepiness.

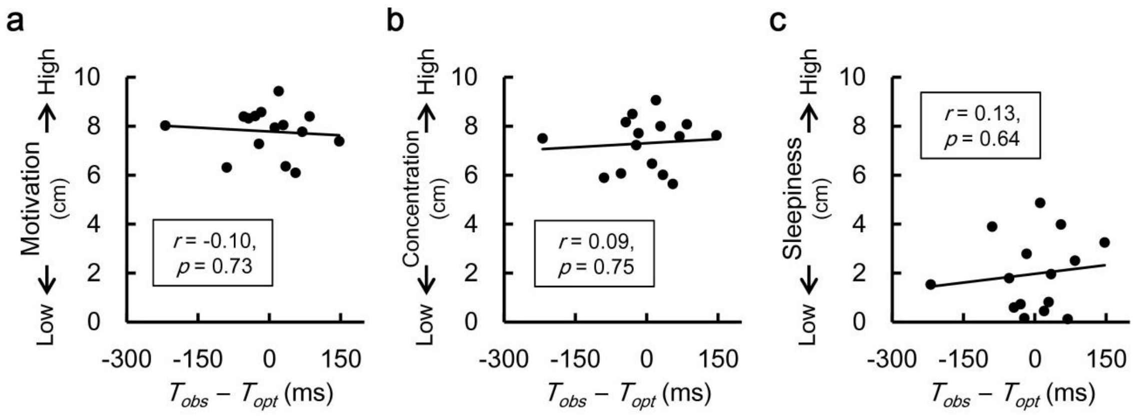

Supplementary Figure 1: No correlation between the motor planning under risk and the levels of motivation (a), concentration (b), and sleepiness (c). Each symbol represents each participant.

2. Inter-personal differences in strategy

Supplementary Figure 2 shows the observed mean response time against the SD of the response time for 12 of 15 participants. The result for the remaining 3 participants is presented in Figure 6. For clarity, we arranged participants in each column (risk-averse, risk-neutral, and risk-seeking) based on the difference between the observed and optimal mean response time in the last 10 blocks (i.e., day 8 & day 9).

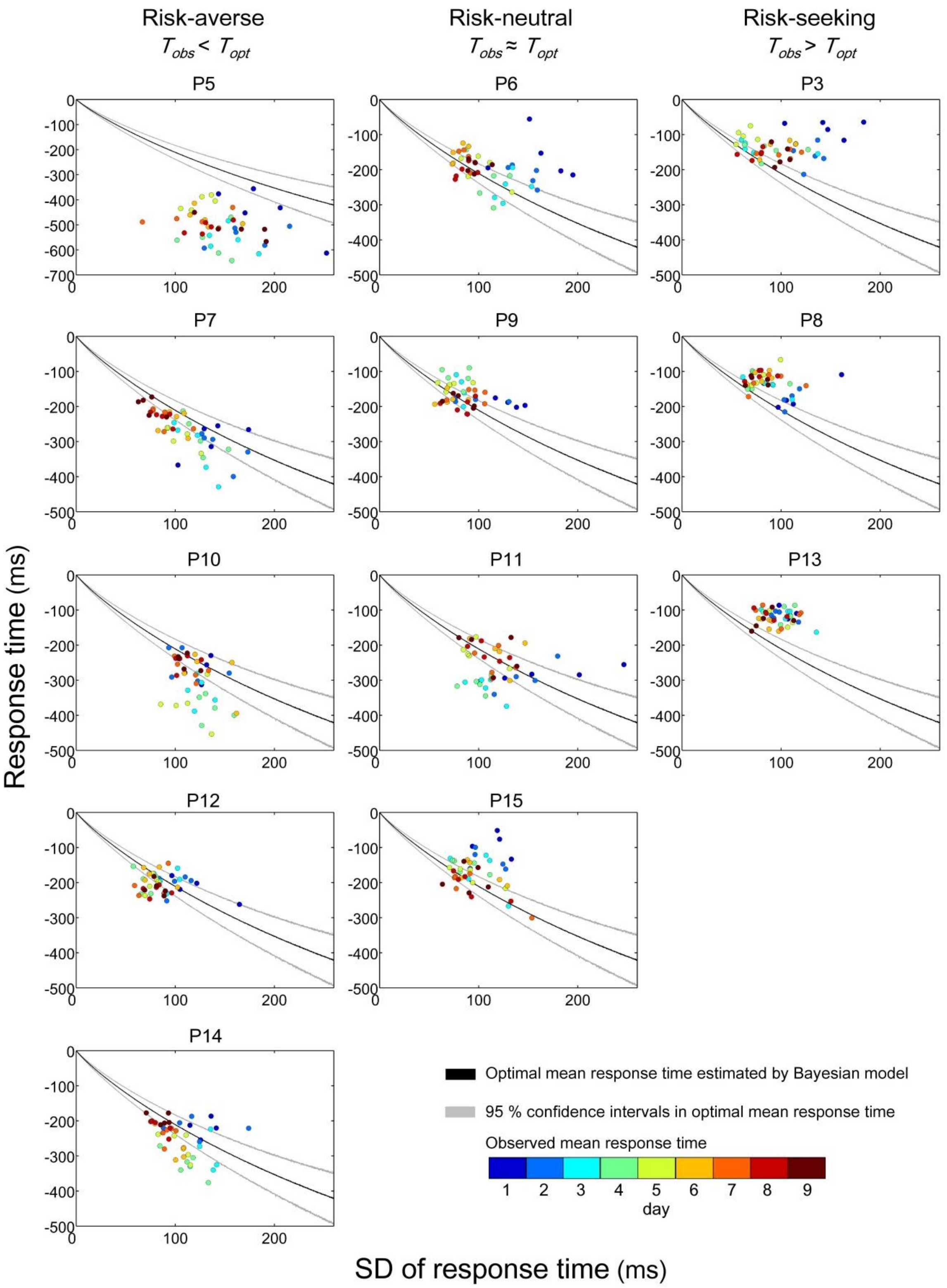

Supplementary Figure 2: In each panel, the observed mean response times for all 45 blocks were plotted against the SD of the response time. The colour scale of the circles shows the day

of the measurements. Black curves indicate the optimal mean response time calculated using the Bayesian model (Equation 2). Grey curves indicate the 95% confidence intervals of the optimal mean response times obtained using a bootstrapping algorism.

3. Consistency of motor planning under risk

We performed a regression analysis between the differences of T_{obs} and T_{opt} from day 1 to day 9. Supplementary Table 1 shows the regression matrix. A slope of a regression line, 95% confidence intervals (CI) of slope, a coefficient of determination (R^2), and P value are plotted.

Supplementary Table 1: Regression matrix of the difference of T_{obs} and T_{opt}.

			$T_{obs} - T_{opt}$ on							
			D2	D3	D4	D5	D6	D7	D8	D9
$T_{obs} - T_{opt}$ on	D1	Slope	1.06	0.97	1.10	0.85	0.80	0.83	0.71	0.70
		95% CI of slope	0.68-1.45	0.52-1.43	0.61-1.59	0.49-1.21	0.48-1.12	0.46-1.20	0.25-1.17	0.41-0.998
		R^2	0.73	0.62	0.65	0.67	0.69	0.64	0.46	0.67
		P value	0.00	0.00	0.00	0.00	0.00	0.00	0.01	0.00
	D2	Slope		0.88	0.99	0.76	0.70	0.75	0.74	0.65
		95% CI of slope		0.61-1.15	0.69-1.28	0.56-0.97	0.52-0.89	0.53-0.96	0.51-0.97	0.52-0.78
		R^2		0.79	0.80	0.83	0.83	0.81	0.79	0.90
		P value		0.00	0.00	0.00	0.00	0.00	0.00	0.00
	D3	Slope			1.08	0.72	0.68	0.78	0.75	0.64
		95% CI of slope			0.94-1.23	0.47-0.98	0.46-0.91	0.60-0.96	0.52-0.98	0.47-0.80
		R^2			0.95	0.74	0.77	0.88	0.79	0.84
		P value			0.00	0.00	0.00	0.00	0.00	0.00
	D4	Slope				0.68	0.62	0.71	0.67	0.58
		95% CI of slope				0.48-0.88	0.42-0.82	0.57-0.86	0.46-0.89	0.44-0.72
		R^2				0.80	0.78	0.89	0.78	0.85
		P value				0.00	0.00	0.00	0.00	0.00
	D5	Slope					0.82	0.88	0.87	0.74
		95% CI of slope					0.57-1.08	0.60-1.15	0.57-1.17	0.52-0.96
		R^2					0.79	0.78	0.75	0.80
		P value					0.00	0.00	0.00	0.00
	D6	Slope						0.98	1.01	0.85
		95% CI of slope						0.73-1.24	0.77-1.25	0.68-1.02
		R^2						0.84	0.87	0.90
		P value						0.00	0.00	0.00
	D7	Slope							0.96	0.81
		95% CI of slope							0.76-1.16	0.68-0.93
		R^2							0.89	0.94
		P value							0.00	0.00
	D8	Slope								0.79
		95% CI of slope								0.65-0.93
		R^2								0.92
		P value								0.00

4. Consistency of distortion in utility function

We performed a regression analysis between the values of the exponential parameter α from day 1 to day 9. Supplementary Table 2 shows the regression matrix. A slope of a regression line, 95% confidence intervals (CI) of slope, a coefficient of determination (R^2), and P value are plotted.

Supplementary Table 2: Regression matrix of the exponential parameter α.

		α on							
		D2	D3	D4	D5	D6	D7	D8	D9
α on D1	Slope	0.68	0.51	0.55	0.60	0.58	0.52	0.35	0.39
	95% CI of slope	0.39-0.97	0.23-0.80	0.09-1.01	0.24-0.95	0.18-0.99	0.07-0.97	-0.11-0.80	0.09-0.70
	R^2	0.66	0.54	0.34	0.50	0.43	0.33	0.17	0.37
	P value	0.00	0.00	0.02	0.00	0.01	0.03	0.12	0.02
D2	Slope		0.72	0.86	0.78	0.88	0.88	0.74	0.67
	95% CI of slope		0.47-0.97	0.42-1.29	0.41-1.16	0.53-1.24	0.50-1.26	0.34-1.14	0.46-0.89
	R^2		0.75	0.58	0.61	0.69	0.66	0.55	0.78
	P value		0.00	0.00	0.00	0.00	0.00	0.00	0.00
D3	Slope			1.25	1.08	0.99	1.20	0.99	0.82
	95% CI of slope			0.95-1.56	0.75-1.41	0.51-1.47	0.88-1.51	0.58-1.40	0.56-1.07
	R^2			0.86	0.80	0.61	0.84	0.67	0.78
	P value			0.00	0.00	0.00	0.00	0.00	0.00
D4	Slope				0.78	0.74	0.91	0.70	0.59
	95% CI of slope				0.53-1.04	0.39-1.09	0.71-1.11	0.37-1.03	0.39-0.79
	R^2				0.77	0.62	0.89	0.62	0.75
	P value				0.00	0.00	0.00	0.00	0.00
D5	Slope					0.97	1.01	0.86	0.70
	95% CI of slope					0.71-1.22	0.77-1.24	0.56-1.16	0.53-0.88
	R^2					0.84	0.86	0.75	0.85
	P value					0.00	0.00	0.00	0.00
D6	Slope						0.91	0.78	0.68
	95% CI of slope						0.62-1.19	0.47-1.10	0.54-0.82
	R^2						0.78	0.70	0.89
	P value						0.00	0.00	0.00
D7	Slope							0.83	0.67
	95% CI of slope							0.59-1.06	0.56-0.79
	R^2							0.82	0.92
	P value							0.00	0.00
D8	Slope								0.71
	95% CI of slope								0.54-0.88
	R^2								0.86
	P value								0.00

5. Model assumption based on Weber's law

We calculated the optimal mean response time based on the model that takes Weber's law into account. Here we call response time as button press time from onset of a start signal (visual cue). In this model, the probability distribution of response time t was defined as a Gaussian distribution with mean T and standard deviation wT, which scaled linearly with a planned response time T with a constant coefficient of variation w[1)] as follows. Supplementary Figure 3a shows an example of distributions when w is 0.05.

$$P(t|T) = \frac{1}{\sqrt{2\pi(wT)^2}} exp\left[-\frac{(t-T)^2}{2(wT)^2}\right] \quad \text{(S1)}$$

The expected gain EG can be calculated by integrating the gain function under Risk condition $G(t)$ over the probability distribution $P(t|T)$.

$$EG(T) = \int_{-\infty}^{\infty} G(t) \cdot P(t|T)dt \quad \text{(S2)}$$

Supplementary Figure 3b shows the expected gain as a function of a planned response time when w is 0.05. We calculated the optimal mean response time T'_{opt} by maximizing the expected gain.

$$T'_{opt}(w) = \underset{T}{\operatorname{argmax}}\, EG(T) \quad \text{(S3)}$$

For $w =$ 0.05, the maximum expected gain is 89 points and the optimal mean response time is 2078 ms (Supplementary Fig. 3b). As shown in Equation S3, the optimal mean response time in proportional variance model can be a function of Weber fraction.

Black curves in Supplementary Figure 3c shows the optimal mean response time T_{opt} calculated from the model based on constant response variance (Eq. 1&2). Note that T_{opt} is a function of the SD of response time (upper horizontal axis). From the obtained relation between T_{opt} and the SD of response time, we calculate Weber fraction, $w = \frac{\sigma}{T_{opt}}$. For example, when σ is 100 ms, T_{opt} can be 2089 ms. For these values, w can be 0.048. When σ is 200 ms, T_{opt} can be 1947 ms and w can be 0.103.

We then calculated the optimal mean response time in proportional variance model T'_{opt} from the obtained value of Weber fraction. Red curves in Supplementary Figure 3c shows T'_{opt} as a function of Weber fraction (lower horizontal axis). As shown in Supplementary Figure 3c, the deviation between T_{opt} and T'_{opt} is larger as Weber fraction or the SD of response time is larger. We show 95% confidence interval (CI) of the SD of response time obtained in the Risk condition, as the gray region. The deviation between T_{opt} and T'_{opt} was 19 ms (1951 ms -

1932 ms) in the upper limit of 95% CI ($\sigma = 190$ ms). The difference between T_{opt} and T_{obs} that we found in the experiment was clearly larger than this deviation (see Fig. 3e).

Furthermore, based on the proportional variance model, we calculated a slope of the regression line between the difference of T_{obs} and T'_{opt} on day 1 and that on day 9. We found it to be a slope of 0.69 (Supplementary Fig. 3d). A regression slope between the difference between T_{obs} and T_{opt} on day 1 and that on day 9 was 0.70 (Fig. 4a).

We also conducted an additional experiment to measure participant's Weber fraction. In this experiment, three participants (P2, P5, and P6) performed the task with four different timing intervals (800 ms, 2300 ms, 3800 ms, and 5300 ms) for 50 trials each. They were instructed to press a button aiming at these intervals. We assumed that participant's response variance σ^2 is a linear function of the planned response time T, $\sigma^2 = (wT + b)^2$. From the response variance and the mean response time data, we estimated w and b. The estimated w were 0.028, 0.033, and 0.037 and b were 0.064, 0.066, and 0.032 for P2, P5 and P6 respectively. For these values of w, the deviations between T_{opt} and T'_{opt} were 1 ms (2160 ms - 2159 ms), 3 ms (2140 ms - 2137 ms), and 3 ms (2125 ms - 2122 ms).

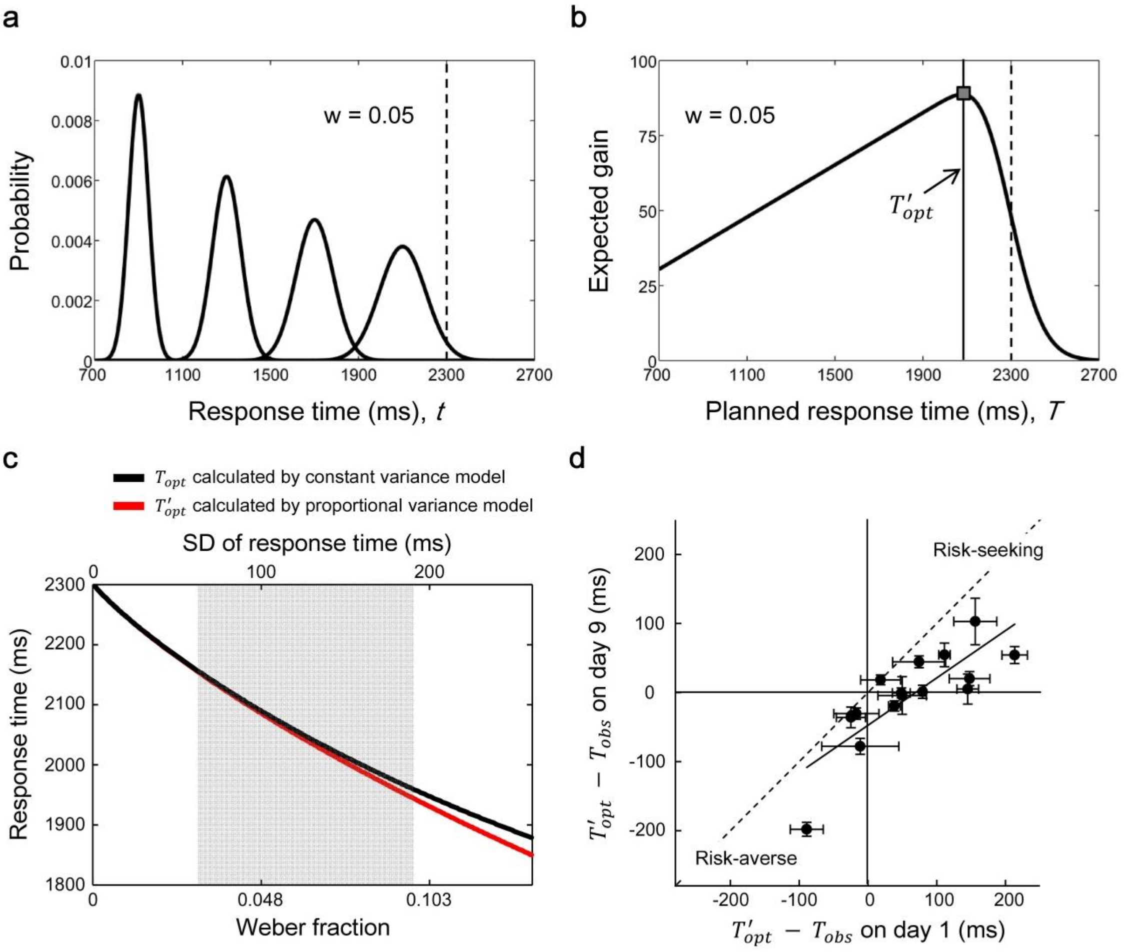

Supplementary Figure 3: (a) The probability distribution of response time with Weber fraction w. The temporal variance is proportional to a planned response time. Here we show an example of distributions when w is 0.05. (b) Expected gain as a function of a planned response time for $w = 0.05$. The vertical line indicates the optimal mean response time. (c) Comparison of 2 models. Black curves represent the optimal mean response time based on constant variance model T_{opt}. This is calculated as a function of the SD of response time (upper horizontal axis). Red curves represent the optimal mean response time based on proportional variance model T'_{opt}. This is calculated as a function of Weber fraction (lower horizontal axis). Gray region indicates 95% confidence interval (CI) of the SD of response time obtained in the Risk condition. From the additional experiment, we obtained three participant's Weber fraction ($w =$ 0.028, 0.033, and 0.037). For these values of w, there are marginal deviations between T_{opt} and T'_{opt}, as shown in Supplementary Figure 3c. (d) Linear regression analysis. A slope of the regression line between T_{obs} and T'_{opt} on day 1 and that on day 9 resulted in 0.69.

1) Jazayeri, M., & Shadlen, M. N. Temporal context calibrates interval timing. *Nat. Neurosci.* **13,** 1020-1026 (2010).
